# Data on triiodothyronine treated peroxisome proliferator-activated receptor-alpha-null mouse hearts using magnetic resonance imaging and magnetic resonance spectroscopy

**DOI:** 10.1016/j.dib.2018.08.009

**Published:** 2018-08-08

**Authors:** Wen Zhang, Michiel ten Hove, Jürgen E. Schneider, Kieran Clarke

**Affiliations:** a Department of Physiology, Anatomy and Genetics University of Oxford, Oxford, United Kingdom; b Radcliffe Department of Medicine, University of Oxford, Oxford, United Kingdom

## Abstract

This data contain left ventricular end-diastolic volumes, end-systolic volumes, stroke volumes, ejection fractions, cardiac outputs, heart rates, phosphocreatine concentrations, adenosine 5’-triphosphate (ATP) concentrations, total creatine concentrations, citrate synthase activities and heart weights for wild-type and peroxisome proliferator-activated receptor-alpha-null mouse hearts without and with triiodothyronine treatment.

**Specifications Table**

**Table:** 

Subject area	Medical Sciences
More specific subject area	Cardiac magnetic resonance imaging and magnetic resonance spectroscopy
Type of data	Figure
How data was acquired	Magnetic resonance imaging, magnetic resonance spectroscopy, HPLC
Data format	Analyzed.
Experimental factors	Male mice received triiodothyronine injection for 7 days.
Experimental features	The effects of triiodothyronine on left ventricular functions, phosphocreatine concentrations, ATP concentrations and total creatine concentrations in peroxisome proliferator-activated receptor-alpha-null mouse hearts perfused with fatty acids free buffer were determined.
Data source location	University of Oxford, United Kingdom
Data accessibility	All data are available with this article.
Related research article	[1] Zhang W, ten Hove M, Schneider J E, Stuckey D J, Sebag-Montefiore L, Bia BL, Radda GK, Davies KE, Neubauer S and Clarke K. Abnormal cardiac morphology, function and energy metabolism in the dystrophic mdx mouse: an MRI and MRS study. J Mol Cell Cardiol. 2008 45:754-60.

**Value of the Data**

• The data can be used to further investigate whether the cross-talk between peroxisome proliferator-activated receptor-alpha and triiodothyronine signaling pathways *in vitro* happens in hearts *in vivo*.
• The data can be used to investigate how peroxisome proliferator-activated receptor-alpha and triiodothyronine regulate mitochondrial contents in hearts.
• The data can be used to investigate the role of peroxisome proliferator-activated receptor-alpha in left ventricular dysfunction in human non-alcoholic fatty liver disease and in pharmaceutical area.

## 1 Data

**Fig. 1:**
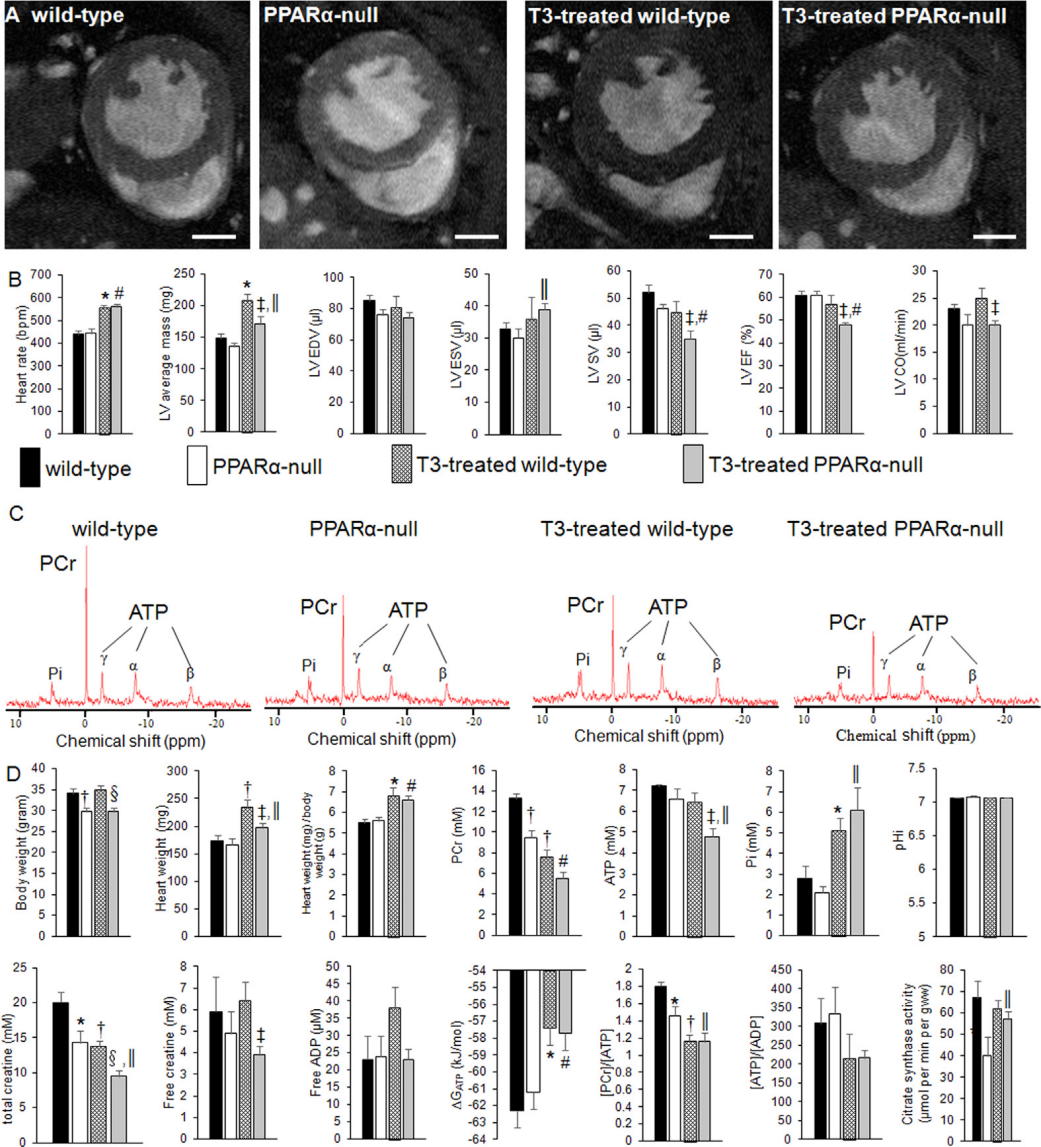
Magnetic resonance imaging and spectroscopy data. A: Representative images at end-diastole. Scale bar = 2 mm. PPARα-null, peroxisome proliferator-activated receptor-alpha-null; T3,triiodothyronine. B: Quantified magnetic resonance imaging data (*n* = 6 for wild-type, *n* = 6 for PPARα-null, *n* = 8 for T3-treated wild-type, and *n* = 6 for T3-treated PPARα-null). LV EDV, left ventricular end-diastolic volume; ESV, end-systolic volume; SV, stroke volume; EF, ejection fraction; CO, cardiac output. Stroke volume = end-diastolic volume – end-systolic volume; ejection fraction = stroke volume/end-diastolic volume; cardiac output = stroke volume x heart rate. C: Representative spectra. Pi, inorganic phosphate; PCr, phosphocreatine; γ, α and β ATP, γ, α and β phosphate groups of adenosine-5’-triphosphate. D: Quantified magnetic resonance spectroscopy, total creatine, and citrate synthase activities data (*n* = 6 for wild-type, *n* = 6 for PPARα-null, *n* = 7 for T3-treated wild-type, and *n* = 7 for T3-treated PPARα-null). ∆G_ATP_, free energy available from ATP hydrolysis; gww, gram wet weight. Data are means ± SEM. * *P* < 0.05 vs. wild-type; † *P* < 0.01 vs. wild-type; ‡ *P* < 0.05 vs. T3-treated wild-type; § *P* < 0.01 vs. T3-treated wild-type, ǁ *P* < 0.05 vs. PPARα-null, # *P* < 0.01 vs. PPARα-null.

Triiodothyronine treatment causes abnormal decrease of left ventricular stroke volumes, phosphocreatine concentrations and ATP concentrations associated with abnormal increase of heart weights and citrate synthase activities in peroxisome proliferator-activated receptor-alpha-null mouse hearts (Fig. 1).

## 2 Experimental design, materials, and methods

Cardiac magnetic resonance imaging, magnetic resonance spectroscopy, total creatine concentrations and citrate synthase activities as previously described [1] were quantified in male wild-type (129S6/SvEv) (*n* = 12), age-matched male peroxisome proliferator-activated receptor-alpha-null mice (129/Sv strain background) (*n* = 12), age-matched triiodothyronine-injected male wild-type (*n* = 15) and age-matched triiodothyronine-injected male peroxisome proliferator-activated receptor-alpha-null mice (*n* = 13) at the age of 8 months old. All the procedures were approved by the Animal Ethics Review Committees, University of Oxford, and by the Home Office, United Kingdom. Triiodothyronine-injected wild-type and peroxisome proliferator-activated receptor-alpha-null mice were injected 3,3׳,5-triiodo-L-thyronine solution (0.2 mg per kg body weight per day) for 7 consecutive days intraperitoneally. Differences between two groups were analyzed by Student׳s *t*-test.
